# Association of bone mineral density with lung function in a Chinese general population: the Xinxiang rural cohort study

**DOI:** 10.1186/s12890-019-1008-2

**Published:** 2019-12-09

**Authors:** Xiang Zeng, Dongling Liu, Xiangmei Zhao, Ling Chao, Yuchun Li, Huijun Li, Wen Li, Lihui Gui, Weidong Wu

**Affiliations:** 1 School of Public Health, Xinxiang Medical University, 601 Jinsui Road, Xinxiang, 453003 Henan China; 2 Henan International Collaborative Laboratory for Health Effects and Intervention of Air Pollution, School of Public Health, Xinxiang Medical University, Xinxiang, 453003 China; 3 Henan Collaborative Innovation Center of Molecular Diagnosis and Laboratory Medicine, Xinxiang Medical University, Xinxiang, 453003 Henan China; 4 Henan Key Laboratory of Medical Tissue Regeneration, Xinxiang Medical University, 601 Jinsui Road, Xinxiang, 453003 China

**Keywords:** BMD, Lung function, General population, Chinese adults, Xinxiang

## Abstract

**Background:**

Bone mineral density (BMD) has been positively associated with lung function in patients diagnosed with respiratory diseases such as chronic obstructive pulmonary disease (COPD) and cystic fibrosis. However, the relationship between BMD and lung function is inconsistent in the general population.

**Methods:**

To investigate the association between BMD and lung function in a Chinese general population, a total of 1024 adults aged 40–70 years old from Qiliying (an industrial polluted exposure area) and Langgongmiao (the reference area with non-industrial pollution) were recruited and underwent BMD and spirometry tests.

**Results:**

Both BMD and lung function levels were lower in the exposed area compared to the reference area. In addition, BMD and lung function levels were also lower in females compared to males. Both Spearman and partial correlation analyses showed that BMD was positively correlated with FVC and FEV_1_. After adjusting linear regression analyses for potential confounding factors, every 0.1 g/cm^2^ drop in BMD was associated with 53.0 mL decrease in FVC and 33.5 mL decrease in FEV_1_.

**Conclusions:**

A reduction of BMD is associated with lower lung function in a general population from China.

## Background

Osteoporosis has gained considerable attention, not only because it can lead to fractures, but also because it is one of the comorbid diseases of respiratory diseases such as chronic obstructive pulmonary disease (COPD) and cystic fibrosis [1–3]. The bone mineral density (BMD) is often used to predict fractures due to its simple and common characteristics [4]. Previous studies have demonstrated that lower BMD is associated with lower lung function in patients diagnosed with respiratory diseases such as COPD and cystic fibrosis [5–8]. However, only few studies investigated the relationship between lung function and BMD in the general population and results were inconsistent in healthy individuals [4, 9–13]. The association between BMD and lung function is therefore uncertain, and whether BMD can be used to predict lung function, or vice versa, remains unclear.

Previous studies have shown that smoking, physical activities, vitamin D intake, and calcium (Ca) supplementation, which are important determinants for BMD and lung function. For instance, several studies found that current smokers have lower BMD and lower lung function, and an increased risk for fractures and airflow obstruction compared to never smokers [14–16]. While physical activity, vitamin D and calcium supplementation have been reported to contribute to the prevention of fractures and the improvement of pulmonary function, although the mechanisms underlying these associations are not fully understood [17–20]. Also socioeconomic status [21, 22], alcohol consumption [23, 24], and dietary habits [25, 26] are independently associated with BMD and lung function, and therefore can confound the association between the two. However, these confounding factors have not all been taken into consideration by previous studies. Moreover, with the more and more serious aging of Chinese populations, the frequency of osteoporosis and osteoporotic fractures will inevitably increase in both males and females. Meanwhile, the air pollution in China is quite serious at the moment, which impairs lung function as harmful fine particulates enter the lungs and cannot be discharged. However, to our knowledge there is lack of studies reporting the relationship between BMD and lung function in the Chinese general population.

Qiliying is an industrial pollution region located in Xinxiang in central China’s Henan province. Investigating individuals exposed to high levels of air pollution, such as adults in Qiliying, may help to identify the factors contribute to BMD and lung function. In the current study, we recruited 1024 adults aged 40 to 70 years from Qiliying (the industrial exposure region), and Langgongmiao (the reference region). This study aims to determine wrist BMD and lung function levels, identify the factors contribute to BMD and lung function, and shed light on the association between BMD and lung function in the Chinese general adult population from an industrial exposure region and a reference region.

## Materials and methods

### Study region and population

**Fig. 1:**
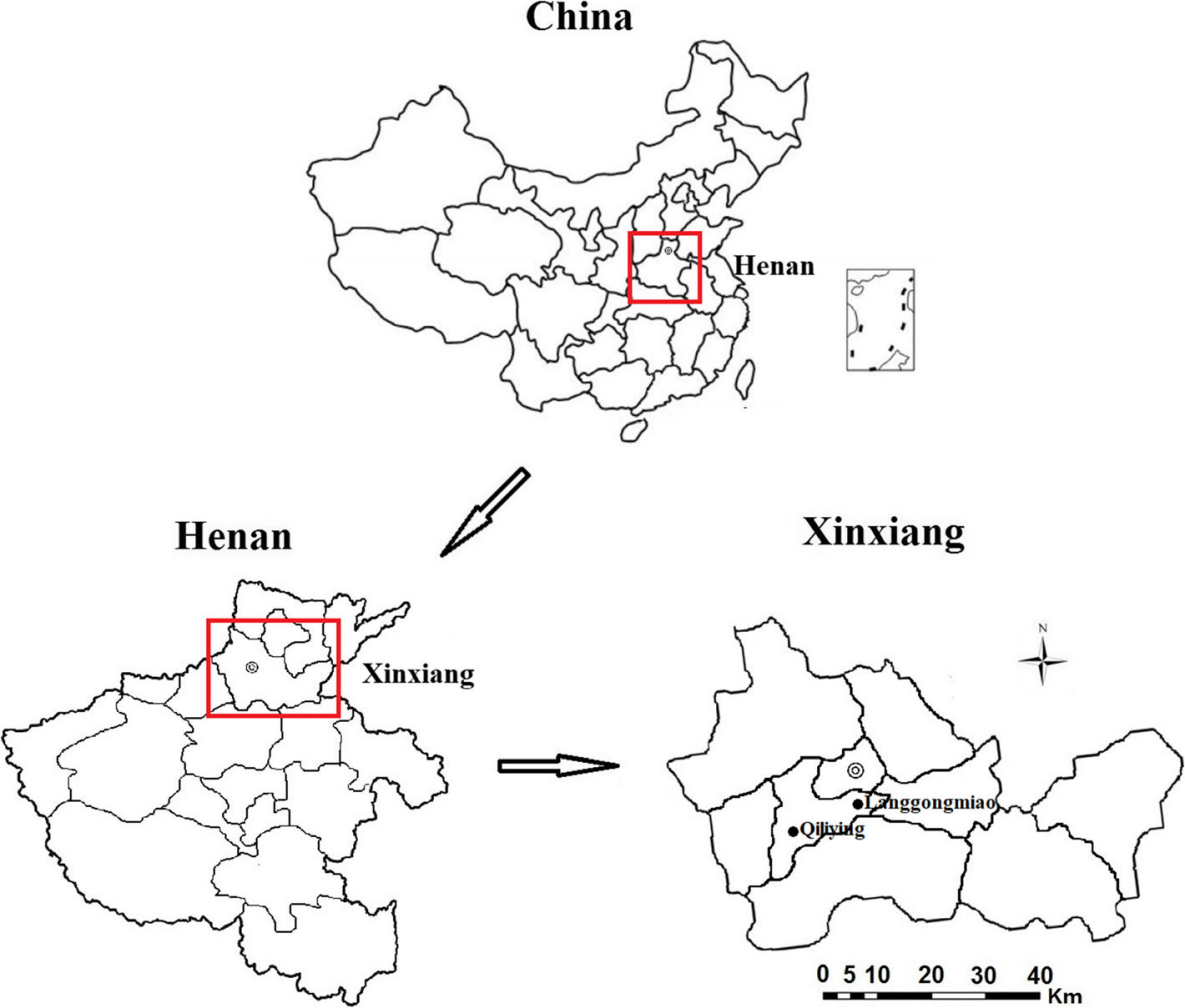
The location of the sampling sites in Xinxiang (Qiliying, the industrial exposure region and Langgongmiao, the reference region)

The survey sites were located in Qiliying (the industrial polluted exposure area) and Langgongmiao town (non-industrial area), Xinxiang county, in the northern part of the Henan Province, central China. Langgongmiao is located 15.6 km to the northeast of Qiliying (Fig. 1). The demographic characteristics, life-styles, eating habits, and traffic conditions are similar in these two areas. The biggest difference between Qiliying and Langgongmiao is that Qiliying is in an industrial polluted area, followed by Qiliying where the family income of residents is higher when compared with Langgongmiao. Langgongmiao was selected as the reference area because it has no industrial pollution and is located upwind of Qiliying. A total of 1024 adults aged 40–70 years were recruited from Qiliying (*n* = 611, all the inhabitants of a village close to industrial factories) and Langgongmiao (*n* = 413, random selection a village of all the inhabitants) during April 2017 and June 2017. The participants completed a general health questionnaire and underwent routine physical examination. The study protocol was approved by the Human Ethical Committee of Xinxiang Medical University, China. All participants provided their written informed consent before enrollment and data collection.

### Procedures and measurements

Each participant completed a face-to-face interview to collect information on demographic characteristics, personal disease history, and lifestyle (dietary and exercise patterns; tobacco and alcohol consumption; and family income and education level) by trained research staff using a standardized questionnaire [27]. Based on this questionnaire, smoking status was categorized into three categories: current smoker, ever smoker and never smoker. Alcohol drinking status was categorized into current drinking, ever drinking, and never drinking. Family monthly income per capita was classified into five categories: < 500 CNY; 500–999 CNY; 1000–1999 CNY; 2000–2999 CNY; > 3000 CNY. Education levels were classified into five categories: Illiteracy; Primary school; Middle school; High school or secondary school; University/College and above. Dietary, nutritional and physical status have also been included in the questionnaire (Additional file 1: Table S1). The weight and height of the participants were measured according to standard methods. Body weight and height were recorded to the nearest 0.1 kg and 0.1 cm, respectively. Participants were required to be standing barefoot and wearing light indoor clothing during the physical examination. Body mass index (BMI, kg/m^2^) was calculated as body weight (kg) divided by height square (m^2^).

### Spirometry

Spirometry was conducted with a portable spirometer (CHEST M.I., INC. HI-801, Tokyo, Japan) following the standardized procedures of the ATS-criteria. Under the guidance of the field physician, the participants were asked to practice a few times before the formal spirometry test until they felt comfortable and mastered the essentials. Forced vital capacity (FVC) and forced expiratory volume in 1 s (FEV_1_) were measured three times. The highest value of the FVC, FEV_1_ and the ratio of FVC to FEV_1_ were used in the analysis.

### Bone mineral density measurement

Wrist dual-energy X-ray absorptiometry (Shenzhen XRAY Electric Co., Ltd., AKDX-09 W-I, Shenzhen, China) was performed to obtain the bone mineral density (BMD) at the wrist. BMD is expressed as an absolute value and as a T-score (standard deviations (SD) of the average sex-specific reference value for healthy young adults) or Z-score (deviation of an age-matched reference population, is commonly used for children, teens, premenopausal women, and men under age of 50). According to the World Health Organization (WHO) criteria, BMD was categorized into three categories: Normal BMD with T-scores between 1.0 and − 1.0; Osteopenia with T-scores between − 1.0 and − 2.5; Osteoporosis with T-scores less than or equal to − 2.5 [28]. In addition, a Z-score larger than − 2.0 indicates that BMD is within the normal range compared to peers and a Z-score less than or equal to − 2.0 indicates that BMD is lower than normal peers.

### Blood sample analyses

Venipuncture was performed by the trained nurses. Blood samples were collected after an overnight fasting and were immediately stored in refrigerator and transported to the laboratory at the School of Public Health (Xinxiang Medical University, Xinxiang, China). Routine blood tests were performed using an automatic hematology analyzer (Sysmex XT-1800i) to obtain blood parameters including hemoglobin, hematocrit, platelet counts, thrombocytocrit, white blood cell count, and eosinophils.

### Statistical analysis

All statistical analyses were performed using SPSS software version 21.0 (IBM Corporation, NJ, USA). Continuous variables are presented as mean ± SD (normally distributed data) or median and interquartile range (skewed distributed data), and were compared using the t-test or chi-square test, respectively. Categorical variables are expressed as numbers and proportions, and were compared using the chi-square test. We proposed the hypothesis that industrial pollutant exposure may have adverse effect on lung function and BMD. Hense, Spearman correlation was used to evaluate the strength of the relationship among lung function indicators (FVC, FEV_1_, FEV_1_/FVC), BMD parameters (BMD, T- and Z-scores), and blood parameters (white blood cell count, hemoglobin, and eosinophils). To correct for the effect of gender on lung function and BMD, these analyses were stratified by gender. Multiple linear regression analyses were performed to determine the independent factors associated with lung function (L) and wrist BMD (g/cm^2^), adjusted for age, gender and height. Finally, we assessed the association between lung function and BMD in univariate, basic (adjusting for age, gender, residential region, height, and BMI) and fully (basic + smoking, drinking, family income, education, hemoglobin, platelet count, eating and exercise habits) adjusted models. Potential confounders were included in the each final model when the *p*-value of the confounder was lower than 0.05. All tests were two-tailed and a *p* <  0.05 was considered as statistically significant.

## Result

### General characteristics of the study population

The demographics of the study population are given in Table 1. When comparing the two regions (Qiliying, the industrial exposure region vs. Langgongmiao, the reference region), we found that there was no significant difference in age, sex composition, height, alcohol consumption, and educational levels. However, weight, BMI, smoke, and family monthly income per capita were higher in Qiliying when compared to Langgongmiao (Table 1). The mean age of males and females were 54.0 and 54.7 years, respectively, with no significant difference. The participants were comprised of 499 (48.7%) males and 525 (51.3%) females. The average BMI was 25.9 kg/m^2^ for males and 25.5 kg/m^2^ for females. The average body fat percentage (BFP) was 26.5% for males and 35.1% for females. Almost half of the males smoked and drank alcohol, while most females neither smoked or drank alcohol. For instance, 99.4% of females never smoked, and 97.9% of females never drank alcohol. Income and educational levels of males were both significantly higher than females (Additional file 1: Table S1).

**Table 1:** Demographic characteristics of the study population (*n* = 1024)

Characteristics	Exposure region (*n* = 611)	Reference region (*n* = 413)	*P*-value
Age (years)	54.18 ± 8.68	54.60 ± 8.31	0.447^a^
Gender, n (%)			0.873^b^
Male	299 (48.9)	200 (48.4)	
Female	312 (51.1)	213 (51.6)	
Height (cm)	162.86 ± 8.31	162.66 ± 10.01	0.728^a^
Weight (kg)	69.36 ± 11.93	67.84 ± 10.94	0.039^a^
Body mass index (BMI, kg/m^2^)	25.96 (23.75, 28.24)	25.47 (23.13, 27.64)	0.032^c^
Smoking, n (%)			0.013^b^
Never smoker	410 (67.1)	283 (68.5)	
Ex-smokers/Former smoker	38 (6.2)	43 (10.4)	
Current smoker	163 (26.7)	87 (21.1)	
Alcohol consumption, n (%)			
Never drink	431	281	0.579^b^
Ex-drinker/Former drinker	23	20	
Current drinker	157	112	
Family monthly income level per capita (CNY), *n* (%)			< 0.001^b^
< 500	117 (19.2)	199 (48.2)	
500–999	245 (40.2)	120 (29.1)	
1000–1999	174 (28.5)	61 (14.8)	
2000–2999	33 (5.4)	22 (5.3)	
> 3000	41 (6.7)	11 (2.7)	
Educational level, n (%)			0.103^b^
Illiteracy	26 (4.3)	25 (6.1)	
Primary school	130 (21.3)	100 (24.2)	
Middle school	246 (40.3)	175 (42.4)	
Secondary school or high school	168 (27.5)	95 (23.0)	
College/university	41 (6.7)	17 (4.1)	
Master and above	0 (0.0)	1 (0.2)	

Exposure region: Qiliying; Reference region: Langgongmiao

^a^Analysis by independent-sample t-test

^b^Analysis by chi-square test

^c^Analysis by Manne-Whitney U test

### Differences in lung function and BMD between gender and region

We found that subjects in Qiliying had lower FVC and FEV_1_, but higher FEV_1_/FVC than those in Langgongmiao (Table 2). Average lung function levels were higher in males compared to females (Additional file 2: Table S2). Although males tended to have relatively larger mean absolute BMD than females, they had lower average T-scores and Z-scores when compared to females (Additional file 2: Table S2). Compared to females, the prevalence of osteopenia was higher in males. There was no significant difference in BMD between subjects from Qiliying and Langgongmiao (Table 2).

**Table 2:** Levels of blood, bone density and lung function parameters in adult population from Qiliying and Langgongmiao in Xinxiang (n = 1024)

Characteristics	Exposure region (n = 611)	Reference region (n = 413)	P-value
Lung function parameters			
FVC (L)	3.12 ± 0.63	3.30 ± 0.78	0.000^a^
FEV_1_ (L)	2.64 ± 0.57	2.72 ± 0.61	0.044^a^
FEV_1_ / FVC (%)	0.84 (0.80, 0.88)	0.83 (0.81, 0.85)	0.000^b^
Bone density parameters			
Bone Mineral Density (BMD)	0.484 ± 0.06	0.488 ± 0.06	0.324^a^
BMD T-scores	−0.029 ± 0.896	− 0.049 ± 1.003	0.730^a^
BMD Z-scores	0.899 ± 1.212	0.919 ± 1.327	0.803^a^
Blood parameters			
Red blood cell count, n (10^12^/L)	4.82 ± 0.47	4.88 ± 0.47	0.068^a^
White blood cell count, n (10^9^/L)	6.01 ± 1.50	6.05 ± 1.47	0.728^a^
Hemoglobin (g/L)	142.98 ± 17.43	142.69 ± 16.84	0.791^a^
MCH	29.65 ± 2.27	29.26 ± 2.19	0.007^a^
MCHC	322.70 ± 15.47	318.00 ± 17.74	0.000 ^a^
Hematocrit (%)	44.27 ± 4.76	44.86 ± 4.67	0.049 ^b^
Platelet count (10^9^/L)	239.19 ± 58.61	252.28 ± 64.05	0.001 ^a^
Thrombocytocrit (%)	0.26 (0.22, 0.30)	0.28 (0.23, 0.32)	0.001^b^
RDW-SD (fL)	13.10 (12.70, 13.60)	13.20 (12.80, 13.80)	0.178^c^
RDW-CV (%)	43.0 (41.0, 45.0)	43.0 (41.0, 46.0)	0.043^b^

Exposure region: Qiliying; Reference region: Langgongmiao. Abbreviation, FVC: forced vital capacity; *FEV*_1_: forced expiratory volume in 1 s; *BMD*: Bone Mineral Density; *BMI*: body mass index; *MCH*: mean corpuscular hemoglobin; *MCHC*: mean corpuscular hemoglobin concentration; *RDW*: red blood cell distribution width. ^#^: 0.05 < *p* < 0.10; ^*^: *p* < 0.05, ^**^: *p* < 0.01

### Lung function and related factors

Both FVC and FEV_1_ levels, regardless of gender, were lower in Qiliying compared to the Langgongmiao region. Both measured FVC and FEV_1_ were higher than their predicted values independently of gender and region. In addition, and FEV_1_ were higher for males than for females independent of the region they came from. Female lung levels were significantly lower in post-menopause than those in pre-menopause in females (Additional file 3: Table S3). In addition, female lung function levels were significant lower in the exposed group than their peers in the reference group (Additional file 4: Table S4). Spearman correlation analyses showed that BMD, hemoglobin, and eosinophils were positively correlated with FVC and FEV_1_ (Table 3). BMD Z-Scores and BFP were negatively correlated with FVC and FEV_1_. Multivariate linear regression analyses were performed to evaluate the factors related to lung function parameters in FVC and FEV_1_. FVC and FEV_1_ were found positively associated with height, intake of fruits and bean products, alcohol consumption, aerobic exercise, but negatively associated with BMI, residence in Qiliying, ingestion of pickles/salted vegetables, and frequent siting state (Table 4).

**Table 3:** Spearman correlation coefficients between lung function, bone density, and blood parameters in males (lower left part table, *n* = 499) and females (upper right part table in gray, *n* = 525), respectively

	FVC	FEV_1_	FEV_1_/ FVC	BMD at wrist	BMD T-Scores	BMD Z-Scores	Age	Height	Weight	BMI	BFP	WBC	Hemoglobin	Eosinophils
FVC		0.976^**^	0.700^**^	0.322^**^	0.290^**^	−0.285^**^	−0.718^**^	0.726^**^	0.191^**^	−0.136^**^	− 0.432^**^	− 0.114^**^	− 0.155^**^	−0.139^**^
FEV_1_	0.946^**^		0.803^**^	0.333^**^	0.301^**^	−0.318^**^	− 0.778^**^	0.721^**^	0.184^**^	−0.146^**^	− 0.452^**^	− 0.130^**^	− 0.159^**^	− 0.148^**^
FEV_1_/FVC	0.348^*^	0.588^**^		0.335^**^	0.327^**^	−0.324^**^	−0.833^**^	0.466^**^	0.114^**^	−0.114^**^	−0.413^**^	− 0.155^**^	−0.179^**^	− 0.146^**^
BMD at wrist	0.256^**^	0.304^**^	0.252^**^		0.972^**^	0.577^**^	−0.371^**^	0.173^**^	0.542^**^	0.482^**^	0.095^**^	0.080	−0.025	− 0.021
BMD T-Scores	0.226^**^	0.269^**^	0.259^**^	0.955^**^		0.618^**^	−0.359^**^	0.151^**^	0.539^**^	0.495^**^	0.126^**^	0.095^*^	−0.032	−0.022
BMD Z-Scores	−0.141^**^	−0.172^**^	− 0.169^**^	0.740^**^	0.805^**^		− 0.419^**^	0.025	0.507^**^	0.562^**^	0.493^**^	0.202^**^	0.115^**^	0.168^**^
Age	−0.636^**^	− 0.789^**^	− 0.787^**^	− 0.297^**^	−0.286^**^	0.251^**^		−0.223^**^	−0.024	0.101^**^	0.447^**^	0.138^**^	0.200^**^	0.196^**^
Height	0.725^**^	0.743^**^	0.340^**^	0.214^**^	0.190^**^	0.017	−0.297^**^		0.335^**^	−0.117^**^	− 0.236^**^	− 0.065	− 0.043	−0.027
Weight	0.354^**^	0.401^**^	0.258^**^	0.614^**^	0.582^**^	0.442^**^	−0.215^**^	0.516^**^		0.855^**^	0.561^**^	0.084	0.027	0.112^**^
BMI	−0.099	0.045	0.132^**^	0.590^**^	0.574^**^	0.514^**^	−0.087	0.034	0.844^**^		0.718^**^	0.133^**^	0.038	0.130^**^
BFP	−0.152^**^	− 0.145^**^	−0.074	0.234^**^	0.253^**^	0.367^**^	0.183^**^	0.033	0.591^**^	0.687^**^		0.198^**^	0.192^**^	0.152^**^
WBC	0.067	0.070	0.052^**^	0.012	−0.023	−0.023	−0.005	0.112^**^	0.134^**^	0.090^*^	0.124^**^		0.172^**^	0.226^**^
Hemoglobin	0.184^**^	0.218^**^	0.209^**^	0.087	0.066	−0.061	−0.221^**^	0.145^**^	0.160^**^	0.083	0.094^**^	0.168^**^		0.123^**^
Eosinophils	0.109^*^	0.107^**^	0.017	−0.009	−0.024	−0.047	− 0.037	0.116^**^	0.078^#^	0.022	0.048	0.304^**^	0.031	

The lower left part of the table is based on data of males; The upper right part of the table is based on data of females. Abbreviation, *FVC*: forced vital capacity; *FEV*_1_: forced expiratory volume in 1 s; *BMD*: Bone Mineral Density; *BMI*: body mass index; *BFP*: Body Fat Percentage; *WBC*: White blood cell count. ^#^: 0.05 < *p* < 0.10; ^*^: *p* < 0.05, ^**^: *p* < 0.01

**Table 4:** Multiple linear regression analysis of factors related to lung function levels in adults (*n* = 1024)

	FVC (L)			FEV_1_ (L)		
	B^a^	β^b^	95% CI for B	B^a^	β^b^	95% CI for B
BMD	0.300	0.156	(0.006, 0.606)^*^	0.216	0.109	(0.001, 0.430)^*^
Region	−0.181	0.015	(−0.212, − 0.151)^***^	−0.096	0.011	(−0.118, − 0.074)^***^
Height	0.074	0.877	(0.072, 0.076)^***^	0.063	0.888	(0.061, 0.065)^***^
Weight	0.031	0.514	(0.028, 0.034)^***^	0.027	0.525	(0.024, 0.029)^***^
BMI	−0.029	0.017	(−0.062, 0.003)^#^	−0.013	0.012	(−0.035, 0.010)
Smoking	−0.015	0.023	(−0.060, 0.031)	−0.005	0.016	(−0.031, 0.026)
Alcohol consumption	0.061	0.023	(0.008, 0.016)^***^	0.059	0.016	(0.027, 0.090)^***^
Educational level	0.001	0.010	(−0.018, 0.020)	0.003	0.007	(−0.011, 0.016)
Family Income	−0.011	0.008	(−0.031, 0.009)	−0.008	0.005	(−0.019, 0.003)
Fruits	0.017	0.007	(0.004, 0.031)^*^	0.012	0.005	(0.002, 0.021)^*^
Bean Products	0.023	0.011	(0.002, 0.043)^*^	0.015	0.007	(0.001, 0.030)^*^
Pickles/salted vegetables	−0.012	0.006	(−0.023, − 0.001)^*^	− 0.008	0.004	(− 0.016, 0.001)^*^
Aerobic exercise	0.049	0.019	(0.012, 0.086)^**^	0.028	0.013	(0.002, 0.054)^*^
Siting state	−0.078	0.034	(−0.145, − 0.012)^*^	−0.046	0.024	(−0.093, − 0.001)^*^

Abbreviation, *FVC*: forced vital capacity; *FEV*_1_: forced expiratory volume in 1 s; *BMD*: Bone Mineral Density; *BMI*: body mass index. Models were adjusted for age, gender and height. B^a^: unstandardized coefficients; β^b^: standardized coefficients. CI: confidence interval; Region: Qiliying vs. Langgongmiao;

^#^: 0.05 < *p* < 0.10; ^*^: *p* < 0.05, ^**^: *p* < 0.01

### BMD levels and related factors

Overall, there was no significant difference in BMD, T-score, and Z-score between subjects from Qiliying and Langgongmiao (Table 2). The only exception was that BMD for males from Qiliying was lower compared to males from Langggongmiao. Absolute BMD levels for males were higher than females independent of the region (Additional file 2: Table S2). In contrast, T-scores and Z-scores for males were lower than females independent of the region. Female BMD levels were significantly lower in post-menopause than those in pre-menopause in females (Additional file 5: Table S5). In addition, female BMD levels were significant lower in the exposed group than their peers in the reference group (Additional file 6: Table S6). Spearman correlation analyses showed that BMI and BFP were positively correlated with BMD, BMD T-scores, and BMD Z-scores (Table 3). Multiple linear regression analyses were performed to assess and identify the factors associated with BMD. These analyses showed that BMD was positively associated with weight and BMI, but inversely associated with age, gender (female only), and body fat percentage (Table 4).

### Associations between lung function and BMD

**Fig. 2:**
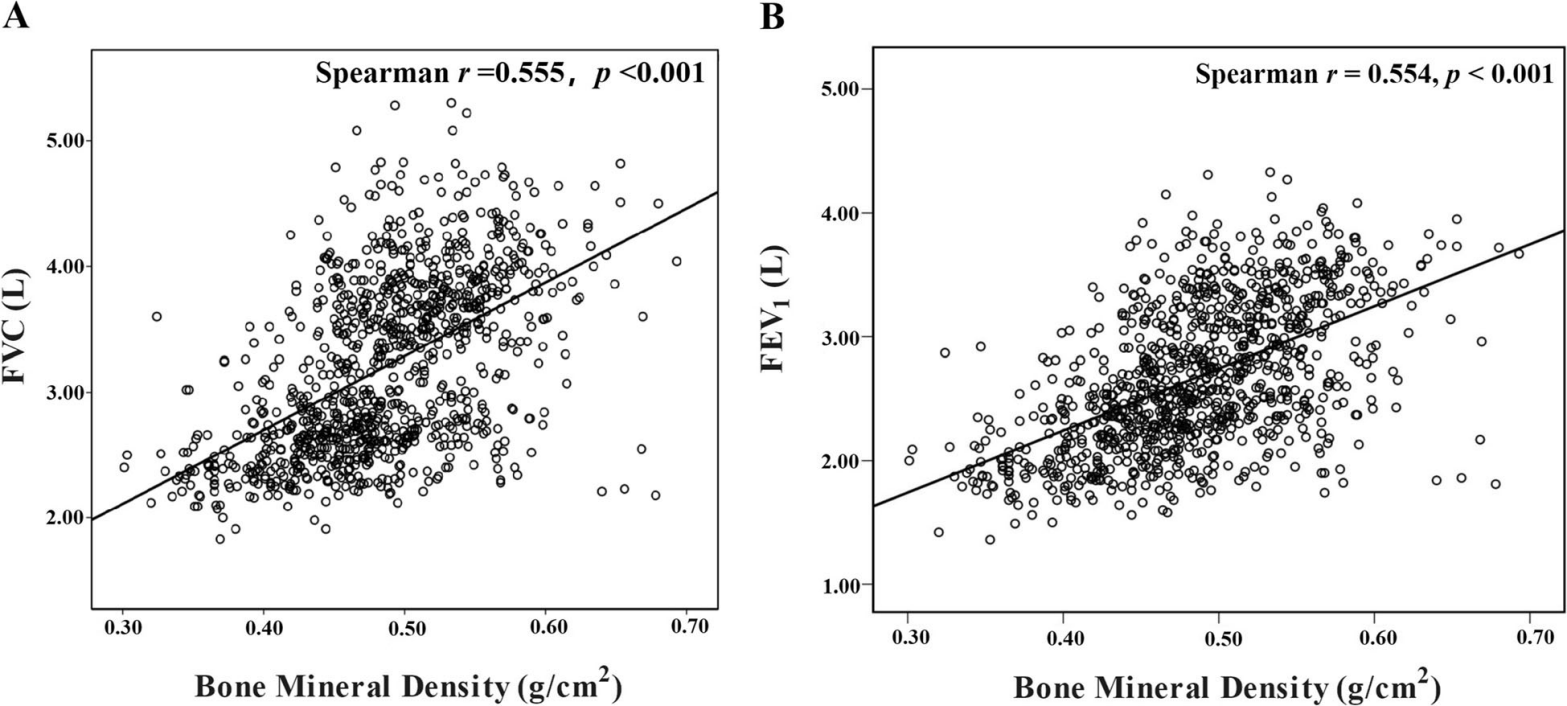
Spearman correlation analyses of the relationship between bone mineral density at wrist and lung function in the Chinese general adult population (*n* = 1024)

The association between BMD and lung function is shown in Fig. 2, Table 3 and Table 5. Spearman correlation analyses showed that BMD was positively correlated with FVC and FEV_1_, respectively (Fig. 2 and Table 3). After adjusting for potential confounders in the multiple linear regression analyses, BMD was still found to be positively associated with FVC and FEV_1_ in the univariate (FVC: B = 5.871; FEV_1_: B = 5.013), basic (FVC: B = 0.550; FEV_1_: B = 0.348) and full models (FVC: B = 0.530; FEV_1_: B = 0.335) (Table 5 and Additional file 7: Table S7). In other words, every 0.1 g/cm^2^ decrease in BMD was associated with 53.0 mL decline FVC and 33.5 mL decline FEV_1_.

**Table 5:** The association between bone mineral density and lung function in adults (*n* = 1024)

	FVC (L)			FEV_1_ (L)		
	B^a^	β^b^	95% CI for B	B^a^	β^b^	95% CI for B
BMD						
Univariate Model	5.871	0.291	(5.301, 6.441)^***^	5.013	0.242	(4.538, 5.489)^***^
Basic Model	0.550	0.164	(0.227, 0.872)^***^	0.348	0.119	(0.114, 0.582)^**^
Full Model	0.530	0.164	(0.208, 0.853)^***^	0.335	0.119	(0.102, 0.568)^**^

Abbreviations: *BMD*: Bone Mineral Density; *FVC*: forced vital capacity; *FEV*_1_: forced expiratory volume in 1 s; *CI*: Confidence intervals; B^a^: unstandardized coefficients; β^b^: standardized coefficients

Basic Models: adjusted for age, gender, group, height, and BMI;

Full Model: adjusted for age, gender, region, height, BMI, smoking, alcohol consumption, family income, education, hemoglobin, platelet count (unit: (10^9^/L)/(1000)), eating and exercise habits

^*^: *p* < 0.05; ^**^: *p* < 0.01^**^; ^***^: *p* < 0.001

## Discussion

The current study assessed the association between wrist BMD and lung function in a Chinese general rural adult population. We found that BMD was positively associated with the lung function outcomes FVC and FEV_1_ after adjusting for potential confounders; every 0.1 g/cm^2^ drop in BMD was associated with 53.0 mL lower FVC and 33.5 mL lower FEV_1_. Age, gender, weight, BMI, and body fat percentage were important factors associated with BMD. Age, gender, height, BMI, place of residence, intake of fruits and bean products, alcohol consumption, aerobic exercise, eating pickles/salted vegetables, and frequent sitting were factors significantly associated with lung function.

We found that both BMD and lung function were negatively correlated with age, BMI, and BFP, but positively correlated with height and weight. The age-adjusted partial correlation coefficients between BMD and FVC or FEV_1_ were 0.475 and 0.469 (*p* <  0.001 for all), respectively. The BMI and BFP-adjusted partial correlations between BMD and FVC or FEV_1_ were 0.373 and 0.400 (*p* < 0.001 for all), respectively. In addition, height and weight-adjusted partial correlations between BMD and FVC, or FEV_1_ were 0.359 and 0.380 (p < 0.001 for all), respectively. After further adjustment for age, BMI, BFP, height and weight, the partial correlation coefficients between BMD and FVC or FEV_1_ were 0.197 and 0.248 (p < 0.001 for all), respectively. Taken together, these results indicate that age, BMI, BFP, height and weight are independent factors involving association between BMD and lung function. Moreover, after performing gender, age, BMI, BFP, height and weight-adjusted partial correlation analyses, the correlation coefficients between BMD and lung function were higher in females than males (p < 0.001 for all). Furthermore, BMD was still positively associated with lung function FVC and FEV_1_ after adjustment for the potential confounders in the multiple linear regression models in the current study.

Although several factors, such as smoking, physical activities, and vitamin D intake, have been considered to play a role in the association between BMD and lung function by previous studies, the results appear inconsistent [4, 11, 13] Other candidate factors such as drinking, socioeconomic status, and eating habits may also contribute to the relationship between BMD and lung function. To assess the effect of these factors on the association between BMD and lung function in the current study, we also adjusted the models for smoking, drinking, physical activities, socioeconomic status, eating habits. We found that low BMD was associated with lung function decline in the China general population.

Many studies have reported the relationship between BMD and lung function in subjects with COPD [5, 6, 29–31] or cystic fibrosis [7, 32–35]. However, inconsistent findings were reported by studies assessing the association between BMD and lung function in the general/healthy population. For example, Jeon et al. did not find a relationship between pulmonary function and BMD in healthy nonsmoking Korean females after adjustment for possible confounding factors in the Korean National Health and Nutrition Examination Survey (KNHANES) [4]. Also, Dennison et al. reported no significant relationship in the Herfordshire cohort study recruiting subjects over 60 years of age after adjusting for body size and other confounders [10], or in both males and females from all region within the KNHANES study [11]. In contrast, Lekamwasam et al. showed that FEV_1_ was positively and independently associated with BMD in 4830 females aged 45–76 years [13] and with hip BMD in 947 men aged 65–76 years from the general population of the United Kingdom [12]. Choi et al. also found that BMD of the lumbar spine and proximal femur were positively correlated with FEV_1_ and peak expiratory flow rate in 98 postmenopausal females compared to 132 premenopausal females from South Korean [9].

The findings of a positive association between BMD and lung function is also supported by the current study, which is unique in adjusting the association for multiple confounding factors such as age, gender, residential area, height, BMI, smoking, alcohol consumption, family income, education, blood parameters, eating and exercise habits. The effect size of the association between BMD and lung function in this study is about 7.5 times bigger comparing to the positive studies mentioned above, which may because that Qiliying and Langgongmiao is in central China where the current background value of environmental pollution, especially for air pollution, is relatively higher compared to the United Kingdom or the South Korean. The mechanism of lung function affecting BMD may be related to hypoxia caused by decreased lung function. Subsequent systemic and gastrointestinal hypoxia can affect the absorption of calcium and vitamin D, thereby affecting bone calcium content, and then leading to the BMD reduction and osteoporosis [36–38]. Conversely, it has been shown that decreased BMD and osteoporosis may cause bone microstructural changes and a decrease in respiratory muscle strength, resulting in thoracic extension limitation and lung function decline [39, 40]. Our previous study showed that chest circumference is a good predictor of lung function in preschool children, which indirectly reflect that the limitation of thoracic extension caused by BMD may lead to a decline in lung function [41]. However, the underlying mechanism of BMD on lung function is still uncertain and needs further study.

The present study has several limitations. First, this is an epidemiological baseline study and a cross-sectional study, which prevents us from making any conclusions about a cause-effect relation between BMD and lung function. Second, only BMD at the wrist was measured and used in this study, and whether wrist BMD can primely represents systemic BMD, such as BMD at the lumbar spine, femur neck, total femur and hip, remains to be determined. Third, vitamin D supplementation and calcium intake status were not investigated in this study. Finally, we had no information about the use, dosage, and duration of corticosteroids which can cause osteoporosis. Nevertheless, to our knowledge this is the first study reporting a positive association between wrist BMD and lung function in general adult population from China.

## Conclusion

Taken together, the results from the present study indicate that a reduction in BMD is associated with worse lung function after adjusting for confounding factors. In addition, males seem to have higher BMD and lung function than females in the Chinese general population aged 40 years and older. In the future, national or regional prospective cohort and controlled studies are necessary to confirm the link between BMD and lung function in the general population.

## Supplementary information

**Additional file 1: Table S1** Demographic characteristics of the study population (*n* = 1024).

**Additional file 2: Table S2** Levels of blood, bone density and lung function parameters in adult population from Qiliying and Langgongmiao in Xinxiang (n = 1024).

**Additional file 3: Table S3** BMD levels between pre-menopause and post-menopause in women

**Additional file 4: Table S4** BMD levels between exposure and reference regions in women.

**Additional file 5: Table S5** Lung function levels between pre-menopause and post-menopause in women.

**Additional file 6: Table S6** Lung function levels between exposure and reference regions in women.

**Additional file 7: Table S7** Multiple linear regression analysis of the association between bone mineral density and lung function (n = 1024).

## Data Availability

Please contact author for data requests.

## Abbreviations

BFP: Body Fat Percentage
BMD: Bone mineral density
BMI: Body mass index
FEV_1_: Forced expiratory volume in 1 s
FVC: Forced vital capacity
MCH: Mean corpuscular hemoglobin
MCHC: Mean corpuscular hemoglobin concentration
RDW: Red blood cell distribution width
WBC: White blood cell count
