# Supplementary material for: Association of bone mineral density with lung function in a Chinese general population: the Xinxiang rural cohort study
Source: BMC Pulm Med. 2019 Dec 9;19:239. doi: 10.1186/s12890-019-1008-2 (PMC6902516; doi:10.1186/s12890-019-1008-2)
Supplement: Supplementary file 1 — Additional file 1: Table S1 Demographic characteristics of the study population (n = 1024). [file 12890_2019_1008_MOESM1_ESM.docx]

**Additional file 1: Table S1. Demographic characteristics of the study population (n = 1024).**

| Characteristics | Male (n = 499) | Female (n = 525) | P-value |
| --- | --- | --- | --- |
| Age (years) | 53.96 ± 8.68 | 54.73 ± 8.37 | 0.148^a^ |
| Height (cm) | 168.92 ± 6.43 | 156.93 ±10.01 | < 0.001^a^ |
| Weight (kg) | 74.08 ± 11.00 | 63.67 ± 9.63 | < 0.001^a^ |
| Body mass index (BMI, kg/m^2^) | 25.91 (23.60, 28.04) | 25.47 (23.32, 28.03) | 0.492^c^ |
| Body fat percentage (%) | 26.50 (23.05, 29.15) | 35.10 (32.17, 37.70) | < 0.001^b^ |
| Smoking, n (%) |  |  | < 0.001^b^ |
| Never smoker | 171 (34.3) | 522 (99.4) |  |
| Ex-smokers/Former smoker | 80 (16.0) | 1 (0.2) |  |
| Current smoker | 248 (49.7) | 2 (0.4) |  |
| Alcohol consumption, n (%) |  |  | < 0.001^b^ |
| Never drink | 198 (39.7) | 514 (97.9) |  |
| Ex-drinker/Former drinker | 42 (8.4) | 1 (0.2) |  |
| Current drinker | 259 (51.9) | 10 (1.9) |  |
| Family monthly income level per capita (CNY), n (%) |  |  | 0.003^b^ |
| < 500 | 135 (27.1) | 181 (34.5) |  |
| 500 - 999 | 168 (33.7) | 197 (37.5) |  |
| 1000 - 1999 | 130 (26.1) | 105 (20.0) |  |
| 2000 - 2999 | 34 (6.8) | 21 (4.0) |  |
| > 3000 | 31 (6.2) | 21 (4.0) |  |
| Education level, n (%) |  |  | < 0.001^b^ |
| Illiteracy | 10 (2.0) | 41 (7.8) |  |
| Primary school | 88 (17.6) | 142 (27.0) |  |
| Middle school | 208 (41.7) | 213 (40.6) |  |
| Secondary school or high school | 156 (31.3) | 107 (20.4) |  |
| College/university and above | 37 (7.4) | 22 (4.2) |  |

Exposure region: Qiliying; Reference region: Langgongmiao.

^a^ Analysis by independent-sample t-test.

^b^ Analysis by chi-square test.

^c^ Analysis by Manne-Whitney U test.
