# Supplementary material for: Association of bone mineral density with lung function in a Chinese general population: the Xinxiang rural cohort study
Source: BMC Pulm Med. 2019 Dec 9;19:239. doi: 10.1186/s12890-019-1008-2 (PMC6902516; doi:10.1186/s12890-019-1008-2)
Supplement: Supplementary file 2 — Additional file 2: Table S2 Levels of blood, bone density and lung function parameters in adult population from Qiliying and Langgongmiao in Xinxiang (n = 1024). [file 12890_2019_1008_MOESM2_ESM.docx]

**Additional file 2: Table S2 Levels of blood, bone density and lung function parameters in adult population from Qiliying and Langgongmiao in Xinxiang (n = 1024).**

| Characteristics | Male (n = 499) | Female (n = 525) | P-value |
| --- | --- | --- | --- |
| Lung function parameters |  |  |  |
| FVC (L) | 3.79 ± 0.46 | 2.64 ± 0.33 | < 0.001^a^ |
| FEV_1_ (L) | 3.12 ± 0.44 | 2.25 ± 0.34 | < 0.001^a^ |
| FEV_1_ / FVC | 0.81 (0.79, 0.85) | 0.85 (0.83, 0.86) | < 0.001^b^ |
| Bone density parameters |  |  |  |
| Bone Mineral Density (BMD) | 0.518 ± 0.52 | 0.455 ± 0.59 | < 0.001^a^ |
| BMD T-scores | -0.184 ± 0.79 | 0.103 ± 1.048 | < 0.001^a^ |
| BMD Z-scores | 0.394 ± 0.917 | 1.395 ± 1.344 | < 0.001^a^ |
| Blood parameters |  |  |  |
| Red blood cell count, n (10^12^/L) | 5.12 ± 0.43 | 4.60 ± 0.35 | 0.001^a^ |
| White blood cell count, n (10^9^/L) | 6.33 ± 1.55 | 5.74 ± 1.37 | 0.006^a^ |
| Hemoglobin (g/L) | 154.31 ± 11.98 | 132.08 ± 14.11 | 0.042^a^ |
| MCH | 30.27 ± 1.59 | 28.76 ± 2.51 | < 0.001^a^ |
| MCHC | 326.40 ± 14.35 | 315.56 ± 16.81 | 0.001^a^ |
| Hematocrit (%) | 47.33 ± 3.82 | 41.84 ± 3.88 | < 0.001^a^ |
| Platelet count (10^9^/L) | 228.15 ± 53.77 | 259.76 ± 63.72 | 0.001^a^ |
| Thrombocytocrit (%) | 0.24 (0.21, 0.28) | 0.28 (0.24, 0.33) | < 0.001^b^ |
| RDW-CV (%) | 13.25 ± 0.86 | 13.55 ± 1.64 | < 0.001^a^ |
| RDW-SD (fL) | 43.0 (41.0, 45.3) | 43.0 (41.0, 45.0) | 0.053^b^ |
| Eosinophils count, n (10^9^/L) | 0.140 ± 0.125 | 0.107 ± 0.096 | < 0.001^a^ |
| Eosinophil percentage (%) | 1.70 (1.10, 2.70) | 1.50 (0.90, 2.30) | < 0.001^b^ |

Exposure region: Qiliying; Reference region: Langgongmiao. Abbreviation, FVC: forced vital capacity; FEV_1_: forced expiratory volume in 1 second; BMD: Bone Mineral Density; BMI: body mass index; MCH: mean corpuscular hemoglobin; MCHC: mean corpuscular hemoglobin concentration; RDW: red blood cell distribution width. ^a^Analysis by independent-sample t-test. ^b^Analysis by Manne-Whitney U test; ^#^: 0.05 < p < 0.10; ^*^: p < 0.05, ^**^: p < 0.01.
