# Supplementary material for: Association of bone mineral density with lung function in a Chinese general population: the Xinxiang rural cohort study
Source: BMC Pulm Med. 2019 Dec 9;19:239. doi: 10.1186/s12890-019-1008-2 (PMC6902516; doi:10.1186/s12890-019-1008-2)
Supplement: Supplementary file 3 — Additional file 3: Table S3 BMD levels between pre-menopause and post-menopause in women [file 12890_2019_1008_MOESM3_ESM.docx]

**Additional file 3: Table S3 BMD levels between pre-menopause and post-menopause in women.**

| Women (n=525) | Pre-menopause (208) | Post-menopause (317) | p value |
| --- | --- | --- | --- |
| BMD | 0.472 ± 0.049 | 0.445 ± 0.062 | <0.001 |
