# Supplementary material for: Association of bone mineral density with lung function in a Chinese general population: the Xinxiang rural cohort study
Source: BMC Pulm Med. 2019 Dec 9;19:239. doi: 10.1186/s12890-019-1008-2 (PMC6902516; doi:10.1186/s12890-019-1008-2)
Supplement: Supplementary file 4 — Additional file 4: Table S4 BMD levels between exposure and reference regions in women. [file 12890_2019_1008_MOESM4_ESM.docx]

**Additional file: 4 Table S4 BMD levels between exposure and reference regions in women.**

| Women (n=525) | Exposure region (n = 312) | Reference region (n = 213) | p value |
| --- | --- | --- | --- |
| BMD | 0.454 ± 0.056 | 0.466 ± 0.061 | <0.05 |
