# Supplementary material for: Association of bone mineral density with lung function in a Chinese general population: the Xinxiang rural cohort study
Source: BMC Pulm Med. 2019 Dec 9;19:239. doi: 10.1186/s12890-019-1008-2 (PMC6902516; doi:10.1186/s12890-019-1008-2)
Supplement: Supplementary file 5 — Additional file 5: Table S5 Lung function levels between pre-menopause and post-menopause in women. [file 12890_2019_1008_MOESM5_ESM.docx]

**Additional file 5: Table S5** Lung function levels between pre-menopause and post-menopause in women.

| Women (n=525) | Pre-menopause (208) | Post-menopause (317) | p value |
| --- | --- | --- | --- |
| FVC (L) | 2.825 ± 0.267 | 2.518 ± 0.283 | <0.001 |
| FEV_1_ (L) | 2.476 ± 0.237 | 2.096 ± 0.289 | <0.001 |
