# Supplementary material for: Association of bone mineral density with lung function in a Chinese general population: the Xinxiang rural cohort study
Source: BMC Pulm Med. 2019 Dec 9;19:239. doi: 10.1186/s12890-019-1008-2 (PMC6902516; doi:10.1186/s12890-019-1008-2)
Supplement: Supplementary file 6 — Additional file 6: Table S6 Lung function levels between exposure and reference regions in women. [file 12890_2019_1008_MOESM6_ESM.docx]

**Additional file 6: Table S6 Lung function levels between exposure and reference regions in women.**

| Women (n=525) | Exposure region (n = 312) | Reference region (n = 213) | p value |
| --- | --- | --- | --- |
| Average menopause time (y) | 49.00 ± 4.06 | 50.01 ± 4.57 | <0.05 |
| FVC (L) | 2.603 ± 0.264 | 2.704 ± 0.312 | <0.001 |
| FEV_1_ (L) | 2.224 ± 0.306 | 2.289 ± 0.315 | <0.05 |
