# Supplementary material for: Association of bone mineral density with lung function in a Chinese general population: the Xinxiang rural cohort study
Source: BMC Pulm Med. 2019 Dec 9;19:239. doi: 10.1186/s12890-019-1008-2 (PMC6902516; doi:10.1186/s12890-019-1008-2)
Supplement: Supplementary file 7 — Additional file 7: Table S7 Multiple linear regression analysis of the association between bone mineral density and lung function (n = 1024). [file 12890_2019_1008_MOESM7_ESM.docx]

**Additional file 7: Table S7** Multiple linear regression analysis of the association between bone mineral density and lung function (n = 1024).

|  | FVC (L) | | |  | FEV_1_ (L) | | |
| --- | --- | --- | --- | --- | --- | --- | --- |
|  | B^a^ | β^b^ | 95% CI for B |  | B^a^ | β^b^ | 95% CI for B |
| Gender | -0.719 | 0.029 | (-0.776, -0.663)^***^ |  | -0.452 | 0.021 | (-0.493, -0.412)^***^ |
| Age | -0.020 | 0.001 | (-0.023, -0.018)^***^ |  | -0.028 | 0.001 | (-0.029, -0.026)^***^ |
| Region | -0.177 | 0.016 | (-0.208, -0.145)^***^ |  | -0.096 | 0.012 | (-0.119, -0.073)^***^ |
| Height | 0.029 | 0.001 | (0.026, 0.031)^***^ |  | 0.028 | 0.001 | (0.026, 0.029)^***^ |
| BMI | -0.047 | 0.018 | (-0.082, -0.013)^**^ |  | -0.026 | 0.013 | (-0.051, -0.001)^*^ |
| Smoking | 0.002 | 0.022 | (-0.042, 0.046) |  | 0.005 | 0.016 | (-0.026, 0.037) |
| Alcohol consumption | 0.056 | 0.022 | (0.013, 0.099)^*^ |  | 0.051 | 0.016 | (0.020, 0.082)^***^ |
| Educational level | 0.007 | 0.009 | (-0.012, 0.025) |  | 0.006 | 0.007 | (-0.007, 0.019) |
| Family Income | -0.008 | 0.008 | (-0.023, 0.007) |  | -0.001 | 0.005 | (-0.012, 0.010) |
| Fruits | 0.019 | 0.009 | (0.000, 0.037)^***^ |  | 0.012 | 0.005 | (0.002, 0.021)^*^ |
| Bean Products | 0.016 | 0.010 | (-0.003, 0.036) |  | 0.015 | 0.007 | (0.001, 0.030)^*^ |
| Pickles/salted vegetables | -0.013 | 0.005 | (-0.023, -0.002)^*^ |  | -0.008 | 0.004 | (-0.016, 0.001)^*^ |
| Aerobic exercise | -0.023 | 0.019 | (-0.059, 0.014) |  | 0.028 | 0.013 | (0.002, 0.054)^*^ |
| Sit state | -0.074 | 0.034 | (-0.138, -0.010)^*^ |  | -0.046 | 0.024 | (-0.093, -0.001)^*^ |
| BMD | 0.530 | 0.164 | (0.208, 0.853)^***^ |  | 0.335 | 0.119 | (0.102, 0.568)^**^ |

Full model of the association between bone mineral density and lung function using multiple linear regression analysis (the details information of full model for Table 5).

^*^: p<0.05; ^**^: p<0.01^**^; ^***^: p<0.001.
